# Influence of Blood–Brain Barrier Integrity on Brain Protein Biomarker Clearance in Severe Traumatic Brain Injury: A Longitudinal Prospective Study

**DOI:** 10.1089/neu.2019.6741

**Published:** 2020-05-27

**Authors:** Caroline Lindblad, David W. Nelson, Frederick A. Zeiler, Ari Ercole, Per Hamid Ghatan, Henrik von Horn, Mårten Risling, Mikael Svensson, Denes V. Agoston, Bo-Michael Bellander, Eric Peter Thelin

**Affiliations:** ^1^ Department of Clinical Neuroscience, Karolinska Institutet, Stockholm, Sweden.; ^2^ Department of Section for Perioperative Medicine and Intensive Care, Department of Physiology and Pharmacology, Karolinska Institutet, Stockholm, Sweden.; ^3^ Section of Neurosurgery, Department of Surgery, University of Manitoba, Winnipeg, Manitoba, Canada.; ^4^ Department of Human Anatomy and Cell Science, Rady Faculty of Health Sciences, and University of Manitoba, Winnipeg, Manitoba, Canada.; ^5^ Biomedical Engineering, Faculty of Engineering, University of Manitoba, Winnipeg, Manitoba, Canada.; ^6^ Centre on Aging, University of Manitoba, Winnipeg, Manitoba, Canada.; ^7^ Division of Anaesthesia, Department of Medicine, and Department of Clinical Neurosciences, University of Cambridge, Cambridge, United Kingdom.; ^8^ Department of Division of Clinical Chemistry, and Karolinska Institutet, Stockholm, Sweden.; ^9^ Department of Molecular Medicine and Surgery, Karolinska Institutet, Stockholm, Sweden.; ^10^ Department of Neuroscience, and Karolinska Institutet, Stockholm, Sweden.; ^11^ Department of Neurosurgery, Karolinska Institutet, Stockholm, Sweden.; ^12^ Department of Anatomy, Physiology and Genetics, Uniformed Services University, Bethesda, Maryland, USA.; ^13^ Division of Neurosurgery, Department of Clinical Neurosciences, University of Cambridge, Cambridge, United Kingdom.; ^14^ Department of Theme Neuro, Karolinska Institutet, Stockholm, Sweden.

**Keywords:** albumin quotient, BBB, NSE, S100B, TBI

## Abstract

Brain protein biomarker clearance to blood in traumatic brain injury (TBI) is not fully understood. The aim of this study was to analyze the effect that a disrupted blood–brain barrier (BBB) had on biomarker clearance. Seventeen severe TBI patients admitted to Karolinska University Hospital were prospectively included. Cerebrospinal fluid (CSF) and blood concentrations of S100 calcium binding protein B (S100B) and neuron-specific enolase (NSE) were analyzed every 6–12 h for ∼1 week. Blood and CSF albumin were analyzed every 12–24 h, and BBB integrity was assessed using the CSF:blood albumin quotient (Q_A_). We found that time-dependent changes in the CSF and blood levels of the two biomarkers were similar, but that the correlation between the biomarkers and Q_A_ was lower for NSE (ρ = 0.444) than for S100B (ρ = 0.668). Because data were longitudinal, we also conducted cross correlation analyses, which indicated a directional flow and lag-time of biomarkers from CSF to blood. For S100B, this lag-time could be ascribed to BBB integrity, whereas for NSE it could not. Upon inferential modelling, using generalized least square estimation (S100B) or linear mixed models (NSE), Q_A_ (*p* = 0.045), time from trauma (*p* < 0.001), time from trauma^2^ (*p* = 0.023), and CSF biomarker levels (*p* = 0.008) were independent predictors of S100B in blood. In contrast, for NSE, only time from trauma was significant (*p* < 0.001). These findings are novel and important, but must be carefully interpreted because of different characteristics between the two proteins. Nonetheless, we present the first data that indicate that S100B and NSE are cleared differently from the central nervous system, and that both the disrupted BBB and additional alternative pathways, such as the recently described glymphatic system, may play a role. This is of importance both for clinicians aiming to utilize these biomarkers and for the pathophysiological understanding of brain protein clearance, but warrants further examination.

## Introduction

Traumatic brain injury (TBI) is one of the most common causes of death and disability,^1^ afflicting ∼10,000,000 people annually.^2^ Unconscious TBI patients deemed to be in need of intracranial monitoring are treated in neurocritical care units (NCCUs).^3^ Here, different modalities are monitored in order to prevent secondary insults that may lead to irreversible deterioration in the already damaged brain.^4–6^ Among these modalities, brain-enriched proteins of tissue fate (i.e., “biomarkers”) are increased in body fluids following brain injury and have become increasingly used in the management of TBI.^7^ The two most studied protein biomarkers include the primarily astrocytic S100 calcium binding protein B (S100B) and the neuronal neuron-specific enolase (NSE). Even though neither is brain specific, both are highly brain enriched and have been associated with a worse intracranial condition and long-term functional outcome following TBI.^8–10^ In fact, some centers event utilize one or both in clinical routine work,^11,12^ hence making these two “biomarkers” of particular interest to the neurotrauma translational research field.

The clearance mechanism of protein biomarkers from brain to blood is not fully understood, but is of importance for clinical interpretation and pathophysiological understanding. The adult central nervous system (CNS) comprises three anatomical barriers, namely: (1) the blood–cerebrospinal fluid (CSF) barrier, (2) the blood–brain barrier (BBB), and (3) the arachnoid barrier.^13^ For simplicity, and to align with the clinical research field, we refer to all of these barriers as “BBB.” In addition, a transport route denoted “the glymphatic system” has recently been described in experimental models.^14^ Although it has been suggested in pre-clinical models that brain-enriched proteins in CSF passively leave the brain through this perivascular “glymphatic” system,^15^ others claim that the BBB integrity plays a more decisive role.^16–18^ In TBI studies, in which the patient likely has concomitant injuries to multiple CNS barriers, the gold-standard strategy for assessing BBB disruption (BBBD) is the CSF to blood albumin quotient (Q_A_).^19–25^ Previous studies of TBI and subarachnoid hemorrhage assessing how serum levels of S100B are associated with Q_A_ have not shown significant correlations.^23,26^ However, these studies have been limited by a lack of longitudinal analyses and high frequency sampling during constant CSF drainage. The trajectory of secondary pathophysiological mechanisms following TBI likely require a high temporal resolution,^3^ multi-compartment monitoring, and modeling to elucidate biomarker and Q_A_ dynamics. In aggregate, how brain-enriched proteins leave the injured brain warrants further research, as it may increase understanding of brain injury pathophysiology in several conditions and improve the utility of biomarkers in clinical decision making.

## Aim

We aimed to assess if, and how, clearance of brain-enriched proteins (S100B and NSE) from brain to blood is affected by BBBD, measured as Q_A_, over time utilizing a high-sampling frequency from multiple compartments in severe TBI patients.

## Methods

The included patients were part of a prospective observational study^27^ undertaken at the NCCU at Karolinska University Hospital (Stockholm, Sweden) between January 1, 2010 and March 1, 2013. Written, informed consent was acquired from next-of-kin. The study was conducted in accordance with the Declaration of Helsinki and Swedish law. Ethical approval was provided by the Stockholm County branch of the Central Ethical Review Board (#2009/1112-31/3), now called the Swedish Ethical Review Authority.

### Sample size calculation

Sample size estimation *a priori* using power analysis was unfeasible because to our knowledge, no assumptions on effect size have been described in the literature using the same approach as in our study. However, using data from our group,^26^ we could estimate our sample sizes for a cross-sectional rather than a longitudinal data set. Aiming for a correlation coefficient *r* = 0.60 between S100B_CSF_ and S100B_blood_, we found the estimated sample size needed to reach 80% power on the 0.05 significance level (one-sided test) using the R^28^ package pwr^29^ to be 15 patients. The effect size estimate was larger than the one previously reported from our group (*r* = 0.45),^26^ but smaller than what has more recently been reported^30^ (*r* = 0.79), leading us to believe that this was a valid effect size assumption. We therefore set out to include >15 study subjects.

### Patient inclusion

Because of the sporadic availability of research personnel, recruitment was made periodically. Patients were not randomized, because no group-specific interventions were conducted. Inclusion criteria for the patients were as follows: (1) age 18–75 years, (2) Glasgow Coma Scale (GCS) score 3–8 (unconscious at admission), and (3) computerized tomography (CT) verified structural intraparenchymal intracranial injury. Exclusion criteria comprised: (1) GCS 3 and bilaterally non-responsive pupils, (2) slit ventricles (because that would preclude the planned patient study management by making external ventricular drain (EVD) insertion impossible), (3) unsurvivable injury, (4) unconsciousness of etiology other than TBI, (5) absence of CT-verified intraparenchymal intracranial injury, (6) other concurrent systemic terminal disorder, and (7) impossibility of follow-up (e.g., being foreign citizens).

### Patient management and sample acquisition

The detailed study setup has been described previously.^27^ Briefly, upon admission, patients received an EVD (Medtronic, Eatontown, NJ). The drain was connected to a four-way stopcock (Multiflo 3, BD, Connecta, Franklin Lakes, NJ), connected to a LiquoGuard^®^ CSF-pump (Möller Medical GmbH, Fulda, Germany) set to a constant drainage velocity of 2 mL CSF per hour. CSF was collected in, and samples were obtained from, the drainage bag in the LiquoGuard system in order to minimize the risk for infections, and freeze blocks were used to keep the CSF cold in order to limit protein degradation. The collected pool of CSF and readily acquired arterial blood were analyzed at 6-and 12-h intervals, for both S100B and NSE. Albumin (plasma and CSF) was analyzed once to twice daily. S100B_blood_ was analyzed by electrochemiluminescence immunoassay (Elecsys, Roche Diagnostics, Basel, Switzerland), albumin_plasma_ was analyzed by colorometric bromocresol purple (BCP)-binding assay and albumin_CSF_ was analyzed by immunoturbitity on a Roche Cobas/Modular platform (Roche Diagnostics, Basel, Switzerland). NSE_blood_, NSE_CSF_, and S100B_CSF_ were analyzed by immunoluminometric assay on a LIAISON XL system (DiaSorin, Saluggia, Italy). S100B_blood_ and S100B_CSF_ were analyzed on different platforms because of local procurements and inability to run CSF samples on the more modern Cobas/Elecsys platforms. Each individual assay has shown robust within- and between-run similarity,^31^ thus enabling longitudinal sampling as employed in this study. Some claim that the different platforms are not entirely interchangeable,^32^ whereas others have found excellent associations between the methods (*r* = 0.932).^31^ It is noteworthy that, to our knowledge, no studies have examined bicompartmental similarities (i.e., between CSF and blood). This is of importance, because absolute between-assay agreement is of greater importance when identical samples from the same compartment are examined on multiple platforms. However, for the scope of this study, the relative relationship was of greater interest. All laboratory assays were performed at the Karolinska University Laboratory in accordance with local guidelines. Aside from this, patients were treated in accordance with local routine at the NCCU, as previously described.^8^

### Clinical parameters

Baseline clinical data were defined and acquired as follows. Clinical variables comprised GCS,^33^ (head and non-head) Abbreviated Injury Scale^34^ (AIS), Injury Severity Score^35^ (ISS), and significant multitrauma,^36^ all of which were acquired upon hospital admission. It is of note that an AIS of 6 at admission is impossible, as it denotes an unsurvivable injury. CT variables upon admission were graded using the Marshall CT Classification,^37,38^ in which classifications V and VI were collapsed into one category (“mass lesion”), as we used only the admission CT. For comparison, we also assessed the Rotterdam^39^ and Stockholm CT scores.^40^ Radiological brain injury progression upon subsequent examination was evaluated employing a similar strategy as in previous work.^8,41^ Glasgow Outcome Scale^42^ (GOS) was assessed as described previously.^27^ In short, a neurorehabilitation board-certified physician (P.H.G.) examined patients 6 months following the TBI. GOS comprises 5 categories: (1) dead, (2) persistent vegetative state, (3) severe disability, (4) moderate disability, and (5) low disability.

### Definitions

We used “BBBD” as a comprehensive term to denote all types of barrier disruption that can be discerned following TBI, including disruption of the anatomical BBB as well as of the blood–CSF barrier.^43^ BBBD was defined as the quotient between CSF and blood albumin (Equation 1), because that constitutes the literature gold standard.^19,24,43^ The reference intervals used for all proteins in the current study were defined using reference intervals stipulated by the Karolinska University Laboratory during the time of patient inclusion or at present (Table 1).^44-47^

**Table 1.** Reference Intervals Used

Variable	Reference interval
Albumin_CSF_ (mg/L)	15-29 years: <260
	≥ 50 years: <400
Albumin_blood_ (g/L)	< 41 years: 36-48
	≥ 71 years: 34-45
Q_A_ (no unit)	15-29 years: <0.006
	≥ 50 years: <0.009
S100B_CSF_ (μg/L)	< 5
S100B_blood_ (μg/L)	< 0.11
NSE_CSF_ (μg/L)	< 13
NSE_blood_ (μg/L)	< 18

Reference intervals were defined as the reference intervals used by the Karolinska University Laboratory during the time of patient inclusion in the study or at present.^44–47^

$$Q_{A}=\frac{albumin_{CSF}}{albumin_{plasma}}$$

### Statistical analysis

Statistical analyses were conducted using R,^28^ through the interface RStudio^®^. Continuous data were presented as mean ± standard deviation (SD) if normally distributed, or else median (interquartile range [IQR]). Categorical data were presented as count (%). In graphical depictions and calculations, biomarker values were converted to log10-transformed values, unless otherwise stated. A *p* value ≤0.05 or a 95% confidence interval (CI), where the range of the CI did not contain the value 0, was considered significant.

Several variables were sampled at different time intervals, resulting in time points without sample overlap. This is demonstrated graphically^48^ (Fig. S1). Following a comparison between linear and locally weighted scatterplot smoother interpolation (Fig. S2), this was compensated for by linear interpolation. Data rows that still retained “missing” values for a certain time point because of consecutive non-overlapping sampling time points were handled by complete case analysis.

Correlations (regular correlations are here referred to as “momentary” correlations) between biomarkers in different compartments and Q_A_ were assessed using repeated measures correlation^49^ on log10-transformed variables in order to obtain linear relationships. Each model was examined with regard to relevant assumptions. The R packages rmcorr^50^ and tidyverse^51^ were used for calculations and graphical depictions. Because biomarker levels in different body compartments might correlate in a time-delayed fashion, we also cross-correlated the original data. We abstained from de-trending or differencing the data because the relationship we examined was the underlying shared time trend. For all, the biomarker sampling time points were subdivided into defined time intervals (“lags”) of 0.5 days (12 h). Multiple measurements occurring within one lag were averaged. Last, cross-correlation was conducted per patient, whereupon results were pooled for all patients and lags. For all cross correlations, original data (i.e., not log10-transformed) were used.

For inferential analysis, we compared a general linear model with correlated errors (general least square estimation, a so-called “marginal model”), and a linear mixed model. For both, the nlme package in R was used.^52^ Independent of analysis type, we modelled a within-patient variance-covariance matrix, because we used repeated-measures data. The variance-covariance matrix was constructed as a time series model, for which we determined stationarity, autocorrelation, and partial autocorrelation. We deemed that the data were stationary using the Kwiatkowski–Phillips–Schmidt–Shin test, and therefore abstained from autoregressive integrated moving average modeling.^53^ The variance-covariance matrix hence consisted of an autoregressive-moving average (ARMA) model. We tested 25 different ARMA combinations of varying complexity, and then chose the optimal ARMA structure based on Akaike Information Criterion. For both S100B and NSE we used a first order autoregressive model with a moving average of 1; that is, ARMA(*p* = 1, q = 1). The resulting models were validated graphically.^54^ For general linear as well as linear mixed-model selection we employed both a step-up^55^ and a top-down strategy.^56^ Random effect structures were evaluated using restricted maximum likelihood-based estimations and fixed effects structure using maximum-likelihood estimations. Model selection was conducted by likelihood ratio tests between nested models and type I *F* tests. *P* values of the final models were generated using Satterthwaite approximations in the R package lmerTest.^56,57^ In all analyses, the dependent variable was the biomarker_blood_ value (log10 transformed). For S100B, the first 12 h were excluded from analysis, because these time points have been associated with an “extracranial peak.”^8,9^

In general, Akaike Information Criterion was lower for the mixed-model design in the top-down strategy, whereas they were equal in the step-up strategy. For S100B, the variance-covariance matrix generated in the linear mixed model was not positive-definite, which is why we chose to use the equivalent marginal model for S100B.^56^ For NSE, we had no such issues, which is why we present results from the linear mixed model. It is noteworthy that results were highly similar independent of linear mixed or marginal model design for each biomarker. Model assumptions were examined graphically with regard to homogeneity of variances, normality of variances, correlation between fitted and observed values, and individual assessment of residual autocorrelation and partial autocorrelation. For NSE, one patient (pat #17) followed a markedly different trajectory than the other patients and also constituted an outlier using the model diagnostics previously described. For validation, the results for NSE were run both including and excluding pat #17, and the results were to a large extent similar, leading us to conclude that the outlier pat #17 did not imply any major alterations with regard to overall conclusions.

## Results

### Demographics

In total, 17 patients were recruited, of which one patient was excluded because no samples of CSF albumin had been obtained. Among included patients, there was no loss-to-follow up. Demographics are depicted both individually (Table 2) and for the whole cohort (Table S1). Patients were predominantly middle-aged males, with a median GCS of 7 (4–7). Approximately 40% of the patients had sustained a significant multi-trauma. However, the majority of patients had a head AIS score of 5, suggesting that a severe cranial trauma constituted the predominant pathology. Of all patients, 31.3% had progression of CT-verified lesions during the study period. On long-term follow-up, half of the patients had an unfavorable outcome (GOS 1–3).

**Table 2.** Individual Patient Demographics

Patient No.	Age	Gender	GCS	Pupils				ISS	Marshall	Rotterdam	Stockholm	Brain injury progression	GOS
					Multi-trauma	HeadAIS	Non-headAIS						
1	55	M	8	1	0	5	1	26	VI	5	3.7	0	4
2	53	F	5	2	0	5	1	25	VI	6	3.6	1	3
4	22	M	7	1	1	4	3	29	VI	6	3.3	0	3
5	23	M	8	0	0	4	0	16	III	4	1.5	0	5
6	20	M	8	2	1	3	2	17	II	3	1.8	1	5
7	38	M	7	0	1	5	3	38	II	3	3.5	0	3
8	25	M	8	0	0	5	0	25	IV	5	2	0	5
9	42	M	3	1	1	5	3	38	II	3	1.5	1	3
10	52	F	3	2	1	4	4	29	III	4	3.5	0	3
11	59	M	7	1	0	5	0	25	VI	4	4.9	0	3
12	62	M	3	0	0	5	0	25	II	3	2	1	1
13	49	M	3	0	0	4	0	16	II	3	1.5	0	5
14	20	M	7	0	0	5	0	25	VI	3	2.5	0	4
15	36	M	7	0	1	4	3	26	III	4	2.8	0	4
16	60	F	4	0	0	5	1	25	VI	4	2.5	0	4
17	48	M	4	2	0	5	0	25	VI	5	3.8	1	1

Demographic data of the patient cohort, depicted as each subject's raw data value. One patient (no. 3) was excluded from all analyses because of a lack of albumin_CSF_ samples.

Clinical data upon admission: age (years); gender: M (male), F (female); GCS (Glasgow Coma Scale Score) 3-15; pupils (pupil responsiveness), 0 = bilateral responsive; 1 = unilateral unresponsive; 2 = bilateral unresponsive; multi-trauma, 1 = yes, 0 = no.

Injury scores upon admission: Head and non-head Abbreviated Injury Scale (AIS), (1) minor, (2) moderate, (3) serious, (4) severe, (5) critical, (6) maximum. Injury Severity Score (ISS) (1–75).

Classification upon admission computed tomography (CT): Marshall, (I) no visible pathology,(II) diffuse injury, (III) “swelling,” (IV) shift, V–VI (“mass lesion”). Rotterdam, classes 1–6. Stockholm, tally based. Brain injury progression: 1 = yes, 0 = no.

Outcome data at 6 months follow-up: Glasgow Outcome Scale Score (GOS): (GOS1) dead, (GOS2) persistent vegetative state, (GOS3) severe disability, (GOS4) moderate disability, (GOS5) good recovery.

**FIG. 1.**
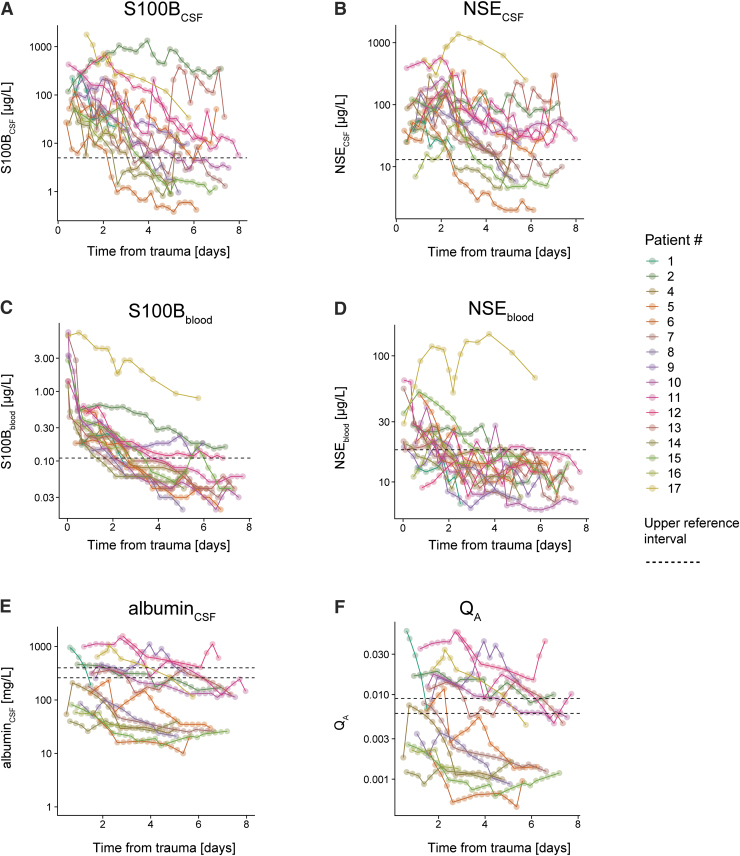
Temporal trajectory of biomarkers and Q_A_ in different compartments. S100B_CSF_ was increased above the reference level **(A)** for all patients following injury and demonstrated a temporal decay. This was reflected in blood **(C)**. For NSE_CSF_ **(B)**, one patient demonstrated normal NSE_CSF_ values following traumatic brain injury (TBI), and even more exhibited normal NSE_blood_ values **(D)**. Albumin_CSF_ was increased among roughly half of the patients **(E)** following the trauma, which was reflected in a pathological Q_A_ **(F)**. Over time, there was a decay in the extent of albumin_CSF_ and a decrease in Q_A_, but interestingly, the extent of damage persisted throughout the whole study period for many patients. Dashed line: upper reference interval as used at the Karolinska University Hospital. Upper reference limit: S100B_CSF_ < 5 μg/L, S100B_blood_ < 0.11 μg/L, NSE_CSF_ < 13 μg/L, NSE_blood_ < 18 μg/L. For albumin_CSF_ and Q_A_ there is an age-dependent reference limit. Upper reference limits for albumin_CSF_: 15–29 years, <260 mg/L; ≥ 50 years, <400 mg/L. Upper reference limits for Q_A_: 15–29 years, <0.006; ≥ 50 years: <0.009. Abbreviations: CSF, cerebrospinal fluid; NSE, neuron-specific enolase; Q_A_, albumin quotient. Color image is available online.

All patients exhibited profoundly increased values of S100B in CSF and blood following trauma (Fig. 1A, C), all higher than the 0.11 μg/L cutoff used to screen if mild TBI patients have an intracranial lesion. A majority of patients also exhibited NSE CSF and blood concentrations above the upper reference intervals of 13 μg/L (CSF) and 18 μg/L (blood) (Fig. 1B, D). Roughly half of the patients demonstrated a disrupted BBB as defined by Q_A_ > 0.006–0.009 (Fig. 1F) upon admission, closely mimicked by CSF levels of albumin >260–400 mg/L (Fig. 1E), but not by blood albumin (Fig. S3). Even though the Q_A_ slope showed a groupwise decreasing trend over time, the study period of ∼1 week was too short to observe normalization of Q_A_ for several patients.

### Correlations between biomarkers and Q_A_

**FIG. 2.**
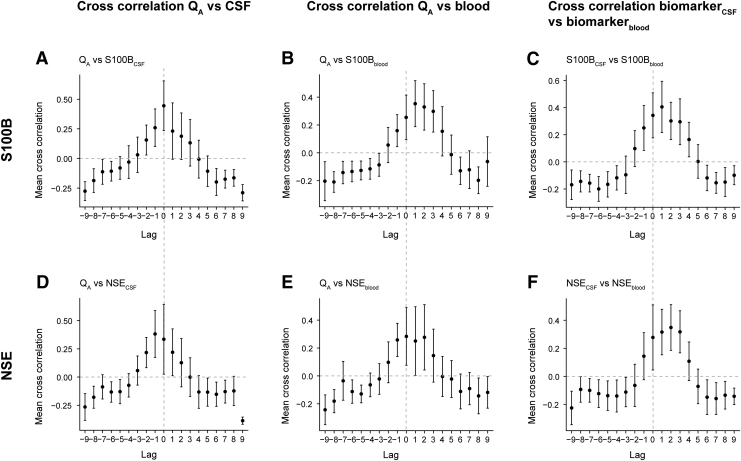
Cross-correlations of biomarkers between compartments and Q_A_. Cross- correlations between BBBD/biomarker_CSF_, BBBD/biomarker_blood_, and biomarker_CSF_/biomarker_blood_ are seen for S100 calcium binding protein B (S100B) (**A–C**) and NSE (**D–F**). S100B_CSF_ and S100B_blood_ had peak correlation at lag 1 **(C)**, indicating that S100B is detected in blood ∼12 h later than in CSF. This could be attributed to the delayed clearance through the disrupted BBB **(B)**. For NSE_CSF_ and NSE_blood_, peak correlation occurred at lag 2, equivalent to 24 h **(F)**, but this could *not* be attributed to either intracranial NSE release **(D)** or any delayed clearance across the BBB of NSE **(E)**. Overall, both S100B and NSE are detected later in blood than they are in CSF, and NSE is the slower of the two proteins. Each lag corresponds to 0.5 days. Dots correspond to mean cross-correlation across a specific lag. Error bars consist of confidence interval (CI), where CI <0 or CI >0 reflect a significant cross-correlation throughout that lag. Dashed lines harmonize lag 0 across all panels. BBB, blood–brain barrier; BBBD, blood–brain barrier disruption; CSF, cerebrospinal fluid; NSE, neuron-specific enolase; Q_A_, albumin quotient.

In order to elucidate the relationship between biomarkers in different compartments and Q_A_, we conducted both regular (here denoted “momentary”) correlation and cross-correlation analyses. Cross-correlation analyses were conducted because clearance from CNS to peripheral blood might occur with a time delay. For cross-correlations, we examined three temporal relationships: (1) correlation between biomarker_CSF_ values and BBBD (Fig. 2A, D); (2) correlation between biomarker_blood_ values and BBBD (Fig. 2B, E); and (3) cross-correlation between biomarker_CSF_ and biomarker_blood_ values (Fig. 2C, F).

There was a strong positive (momentary) correlation between S100B_CSF_ and S100B_blood_ (ρ = 0.667, CI 0.602–0.723) as well as Q_A_ and S100B_blood_ (ρ = 0.668, CI 0.598–0.728). Cross-correlations for S100B showed that there was an immediate correlation between BBBD and S100B_CSF_ (Fig. 2A), whereas there was a lagged relationship between S100B_CSF_ and S100B_blood_ (Fig. 2C). The lag was attributed to the identically lagged relationship between BBBD and S100B_blood_ (Fig. 2B). Hence, S100B exhibited a delayed release to blood, which occurred concomitantly to a delayed leakage across the BBB.

There was also a positive (momentary) correlation between NSE_CSF_ and NSE_blood_ (ρ = 0.436, CI 0.343–0.521) as well as Q_A_ and NSE_blood_ (ρ = 0.444, CI 0.345–0.533). NSE cross-correlations demonstrated a negative lag between BBBD and NSE_CSF_ (Fig. 2D) indicating that NSE_CSF_ levels increased more swiftly than the BBBD occurred following an injury. NSE_CSF_ and NSE_blood_ (Fig. 2F) were positively lagged against one another, meaning that there is a period of delay before NSE can be detected in blood. This phenomenon could not be attributed to the relationship between BBBD and NSE_blood_ (Fig. 2E), because these exhibited a peak correlation at lag 0; that is, immediate to one another. Hence, there was a discrepancy between NSE_CSF_ and NSE_blood_ lags (+2), not accounted for by the other cross correlations (-1 and 0 lags respectively). Further, this means that the delayed NSE clearance did not seem to be a consequence of a delayed intracranial NSE *de novo* release; that is, higher CSF levels (Fig. 2D), or because of a slower clearance across the disrupted BBB (Fig. 2E, lag 0–2).

In summary, a measured blood biomarker concentration is presumably a reflection of both momentary and delayed brain clearance, as well as the extent of the different disintegrated CNS barrier components. For momentary correlations, there was a stronger correlation coefficient for S100B than for NSE. Generally, there were only small differences between the two biomarkers over the different cross-correlations. However, some noteworthy disparities were found. NSE exhibited fewer lags with significant cross- correlations (Fig. 2D–F) than S100B (Fig. 2A–C). Hence, NSE overall demonstrated a less robust pattern than did S100B, which is why the NSE results should be interpreted cautiously. Moreover, in contrast to S100B_blood_, the disintegrated barrier cannot solely explain the temporal pattern of NSE clearance. This is manifested throughout lag 0–2 (equivalent to 0–24 h) (Fig. 2E), where there are, however, small differences in mean cross-correlation and the CIs are very broad, which is why these findings should be interpreted with caution.

### Modelling of biomarker clearance from CSF to blood

**FIG. 3.**
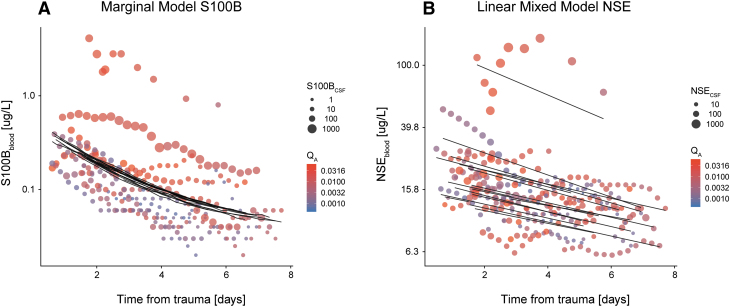
Graphical depictions of clearance models for S100 calcium binding protein B (S100B) and NSE. A marginal model (general least square estimation) was conducted for S100B **(A)** and a linear mixed model was conducted for NSE **(B)**. Time from trauma, S100B_CSF_, and Q_A_ predicted S100B_blood_ in an additive model **(A)**. In contrast, only time from trauma predicted NSE_blood_ **(B)**. For both panels, the size of the circles denotes the biomarker_CSF_ value, whereas the color gradient represents the value of Q_A_. The black lines are the fitted values in each model. CSF, cerebrospinal fluid; NSE, neuron-specific enolase; Q_A_, albumin quotient. Color image is available online.

In order to inferentially determine the importance of Q_A_ for blood biomarker levels, we conducted a generalized least square linear model (marginal model) for S100B (Fig. 3A, Table 3) and a linear mixed effects model for NSE (Fig. 3B, Table 3).

**Table 3.** Inferential Models of S100B and NSE Clearance from CSF to Blood

Marginal model of S100B clearance			Linear mixed model of NSE clearance		
Variable	Estimate	p value	Variable	Estimate	p value
Intercept	-1.13	<0.001	Intercept	1.36	<0.001
Time from trauma	-3.82	<0.001	Time from trauma	-0.0511	<0.001
Time from trauma^2^	0.665	0.023			
S100B_CSF_	0.0247	0.008			
Q_A_	-0.0676	0.045			

*Generalized linear model (marginal model) analysis showing how time from trauma, Q_A_, and S100B_CSF_ were independent predictors of S100B_blood_*. In all analyses, the dependent variable was S100B_blood_. The variables S100B_blood_, Q_A_, and S100B_CSF_ were log10-transformed. Time from trauma was used as a polynomial term with a degree of 2. The underlying correlation structure was modelled as an ARMA(1,1) process, for which the error term depends on the AR component ϕ and the MA component θ. These were estimated to be: ϕ = 0.976 (CI: 0.955–0.987) and θ = 0.563 (CI: 0.456–0.654).

*Linear mixed model analysis showing how time from trauma was an independent predictor of NSE_blood_*. In all analyses, the dependent variable was NSE_blood_. The variable NSE_blood_ was log10-transformed. Random effects of the model were a random intercept (patient) and a random slope (time from trauma). The underlying correlation structure was modelled as an ARMA(1,1) process, for which the error term depends on the AR component ϕ and the MA component θ. These were estimated to be: ϕ = 0.637 (CI: 0.454–0.768) and θ = 0.621 (CI: 0.522–0.704).

AR, autoregressive; ARMA, autoregressive moving average; CI, confidence interval; CSF, cerebrospinal fluid; MA, moving average; NSE, neuron-specific enolase; Q_A_, albumin quotient; S100B, S100 calcium binding protein B.

All models used time from trauma and CSF biomarker levels as covariates together with a pre-determined correlation structure, thereby accounting for the longitudinal study design. Interestingly, there were notable differences between the biomarkers. For S100B (Table 3, Fig. 3A), S100B_blood_ values could be modeled as a function of time from trauma, S100B_CSF_, and Q_A_, all of which were significant. As can be seen in Figure 1C, the relationship followed a curvilinear slope, making the quadratic time from trauma term significant as well. The finding that Q_A_ is a predictor of S100B_blood_ suggests that the extent of BBBD is related to the levels of S100B in blood.

In contrast, for NSE (Table 3, Fig. 3B) only time from trauma emerged as a significant independent predictor, not NSE_CSF_ or Q_A_. These findings were robust even when checked for more complex statistical structures, such as interaction effects. In aggregate, these findings, and the contrast between the biomarkers, indicate that BBBD might affect biomarker clearance from CSF to blood differently for different proteins.

## Discussion

We present a high temporal resolution, multi-compartment, prospective biomarker monitoring study conducted after severe TBI. We examined how BBBD, measured as Q_A_, affects brain clearance of the two most studied protein biomarkers in TBI. We found clear correlations between the blood biomarker values and Q_A_, but coefficients were notably larger for S100B. We also found a cross-correlation between the CSF biomarker and the blood biomarker levels for both S100B and NSE, indicative of a slightly delayed/”lagged” clearance from CSF to blood that for S100B, but not NSE, covaried with a delayed clearance across the BBB. Finally, regression modeling demonstrated that for S100B, time from trauma, S100B_CSF_, and Q_A_ were important contributors to S100B_blood_. In contrast, only time from trauma emerged as a significant predictor of NSE_blood_. We suggest that one reason for the discrepancies between the two biomarkers is that they are cleared differently, with S100B being cleared through a disrupted BBB unlike NSE, but this warrants further examination.

### The disrupted BBB in TBI

We assessed how a trauma-induced BBBD affects brain biomarker concentrations in blood. Even though some radiological techniques have been proposed to quantify BBBD,^58,59^ Q_A_ remains the gold standard assessment,^19–25^ because the 67 kDa protein albumin lacks intracranial synthesis,^19^ is not catabolized in the CNS,^60^ and has a 200 times lower concentration in CSF compared with serum.^19^ We found that whereas Q_A_ on a group basis had a decreasing trend over the 1st week, patients who had normal Q_A_ values at study onset maintained normal values throughout the study period and vice versa. Kleindienst and coworkers also found persistently deranged Q_A_ values in a cohort of TBI and subarachnoid hemorrhage (SAH) patients.^23^ In contrast, Bellander and coworkers found a steep normalization of Q_A_ within the 1st week after TBI,^26^ congruent with data from Blyth and colleagues.^20,21^ This indicates that the BBB is likely more affected by the primary injury than secondary insults, and that BBBD may persist for at least 7 days, or as some have suggested, even longer.^61^ Consistently, some patients did not exhibit any signs of BBBD in our study. The underlying reason for this cannot be causally determined by this work because of the small sample size, but we hypothesize that differences in the intracranial injury panorama is a probable explanation. This finding further highlights the necessity of individualizing treatment following TBI. Finally, we noticed that Q_A_ and albumin_CSF_ mimicked each other closely. Therefore, albumin levels in CSF are presumably enough to assess the BBBD, although this still necessitates CSF sampling, which is not always feasible in the clinical setting.

### Correlation analyses of biomarkers and Q_A_

We found that S100B_blood_ was momentarily correlated to S100B_CSF_ and Q_A_, in accordance with previous studies.^20,21,26,30,59^ Others have acquired contrasting results,^23,26^ possibly because of different analysis strategies. We also noted that momentary NSE_blood_ levels were correlated with NSE_CSF_ and Q_A_, something that has been shown in non-TBI studies,^60,62^ whereas a previous TBI study did not show this association.^26^ For both biomarkers, we noted similar correlation coefficients between CSF:blood and blood:Q_A_. This implies that a third covariate, most likely time from trauma, inferred these values. We therefore performed cross-correlations, to elucidate the underlying share time trend of the biomarker correlations.

In our cross-correlations, we noted that both S100B and NSE exhibited peak CSF:blood cross correlations at lags >0, suggesting that both biomarkers have a partially delayed clearance from CSF to blood. Previous studies examining S100B_serum_ and S100B_CSF_ correlation have found a delay varying between 0–24 h^30^ and 48 h.^63^ Our S100B CSF:blood cross-correlation exhibited a lag of ∼12 h, corresponding to the delay seen between Q_A_ and S100B_blood_. Hence, the CSF:blood and the Q_A_:blood pattern mimicked each other, presumably indicating that there is a brief initial accumulation of S100B in CSF before clearance through a disrupted BBB. This constitutes a tentative pathway for S100B clearance to peripheral blood. In contrast, NSE exhibited a more delayed peak in cross-correlations than S100B (∼ 24 h). This is important, because a brain biomarker should confer accurate and timely information if it is to aid in clinical decision making. Moreover, in the three cross- correlations performed on NSE, *de novo* release of NSE_CSF_ seemed to occur more swiftly than BBBD, and BBBD occurred simultaneously to NSE_blood_ detection. Hence, there was a delayed clearance of NSE from CSF to blood, that was *not* mimicked by either CSF:Q_A_ or Q_A_:blood cross-correlations, which was the case for S100B. Data were scarce for all of these observations, but if true, this means that neither delayed BBB clearance nor delayed *de novo* release of NSE could explain the delayed clearance of NSE_CSF_ to blood. Hence, there is a weak but interesting signal suggesting that intracranial NSE accumulates and thereafter is cleared through a route other than the BBB. One tentative clearance pathway is the experimentally described glymphatic system,^14^ suggested to be a clearance route for both NSE and S100B.^15,64^ Another possible route includes the recently discovered lymphatic vascular network surrounding dural sinuses, which drains in deep cervical lymph nodes and subsequently blood, with clearance of proteins from the brain interstitium.^65,66^ The strong momentary correlations we see between CSF and blood suggests a relatively direct pathway in these glymphatic/brain lymphatic routes, which in the case of NSE is seemingly independent of BBBD. However, it should be noted that both the glymphatic and brain lymphatic vasculature have currently only been sufficiently studied in animal models. Some promising work using magnetic resonance imaging favors the existence and importance of the glymphatic system in humans,^67,68^ but larger human studies and functional characterizations are still warranted.

### BBB disruption predicts S100B but not NSE concentrations in blood

For inferential analysis, we employed marginal and linear mixed modelling. For S100B_blood_, we found that Q_A_, S100B_CSF_, and time from trauma were significant independent predictors. This indicates not only that S100B is dependent on the BBB for clearance to peripheral blood, but also that S100B_blood_ cannot be regarded solely as a marker of BBB integrity as has been suggested.^20,69^ Whereas some claim that cerebral S100B originates from the astrocytic foot processes that enfolds the BBB,^30^ our data rather indicate that S100B_blood_ is a reflection of several factors of which merely one is the extent of BBBD. In a situation in which the BBB is intact,^69^ it is therefore theoretically possible that the S100B_blood_ value is falsely low, as this biomarker will then to some extent be “trapped” in the CSF. Applying a similar approach to NSE, we found that time from trauma was retained as the only significant independent variable in the model. This means that neither NSE_CSF_ nor Q_A_ were predictive of NSE_blood_. Our findings cohere with one previous study that did not find any increase of NSE following chemical/osmolytic BBBD.^70^ In summary, there was a clear relationship between Q_A_ and S100B, whereas for NSE there was not.

Both our cross-correlation and marginal/linear mixed model analyses hence indicate that S100B and NSE might be cleared differently from CSF to peripheral blood. There are, however, important differences in biochemical, kinetic, and/or pathophysiological properties between the two proteins, potentially confounding our results. Biologically, NSE is larger (39 kDa)^71,72^ than S100B (9–14 kDa),^73^ implications of which could be a swifter movement across body compartments for small proteins and a longer serum half-life for larger proteins. Accordingly, NSE has been shown to exhibit an “effective half-life” of up to 48–72 h in serum compared with 24 h for S100B,^74^ following severe TBI. This would give a slower decline of NSE_blood_ values than S100B values. We compensated for this by employing an ARMA variance-covariance structure to our model; however without finding any predictors of NSE_blood_ other than time from trauma. Next, there are different sources for S100B and NSE throughout the body. S100B is present primarily in astrocytes,^75^ but extracranially also in muscle, bone, cartilage, adipose tissue, and melanocytes.^9,76^ The extracranial component is primarily of importance in multi-trauma patients during the first 12 h following the trauma.^8,9^ We excluded the first 12 h from analysis for S100B and monitored patients for a week, which would result in a very limited contribution of extracranial S100B to our model. In contrast, whereas NSE intracranially locates primarily to neurons,^9^ extracerebral NSE emanates from, among other sources, erythrocytes.^9,77^ Hence, extracerebral NSE is a confounding factor following both multi-trauma and hemolysis.^9^ Importantly, there is no clear period after trauma when this is important, but rather this potential confounder spans the entire data material and contributes a latent data noise. In summary, although S100B and NSE seem to have different clearance routes from the injured brain, there are differences between the proteins, which may have influenced our results.

### Limitations

The major limitation in this study relates to the inclusion rate (inclusion was dependent on senior author presence) and the consequently small study sample size. Moreover, there are other factors that may be associated with brain biomarker clearance, such as brain injury severity or type of injury sustained, but these correlations necessitate a larger sample size to study. Conversely, the data was prospectively collected with a uniquely high temporal resolution with constant CSF drainage speed; therefore making this study highly relevant in term of brain protein clearance. Another limitation is that the CSF at time point 0 in our models constituted a pool of CSF collected during the preceding 6 h; therefore, the lag times could be slightly longer than indicated in the cross-correlations, although we believe the current protocol using the CSF pump is the most delicate way to extract CSF for serial sampling using an enclosed system. Further, in animals, it has been shown that acetazolamide treatment, CSF drainage, and sleep deprivation inhibit the glymphatic system.^15^ Although no patients received acetazolamide, and CSF drainage speed was constant in our study, all patients exhibited different levels of induced sedation, which in theory could affect glymphatic clearance, but how this may affect biomarker levels in humans warrants further research. In spite of being a gold standard metric for BBBD, Q_A_ is age dependent,^19^ which is a source of error across all studies using it. Moreover, for TBI patients, intraventricular hemorrhage^26^ and administration of large amount of intravenous fluids could both affect Q_A_. For the latter, we propose that CSF albumin alone might be an adequate surrogate for Q_A_ (and therefore BBBD). Finally, in the injured brain, multiple factors are in play, and it is possible that CNS half-life is discrepant from blood half-life of certain proteins. We do not account for this in our models, and in order to do so, one needs to employ more extensive pharmacokinetic modeling, which will be the aim for future studies that are warranted. Another amenable avenue is radiological techniques^67,68^ using biomarker-specific contrast-enhancement combined with sampling techniques similar to ours in order to elucidate discrepancies between BBB and other plausible clearance pathways.

## Conclusion

Blood biomarker levels of S100B and NSE correlate momentarily with BBB integrity, measured as Q_A_. However, longitudinally, this relationship was stronger for S100B than for NSE. Upon inferential modeling, blood levels of S100B were dependent on Q_A_, which we could not observe for NSE. Although this could be the result of underlying differences between the two proteins such as hemolysis of NSE, it indicates that S100B and NSE might be cleared differentially from the injured CNS, which is novel and important for brain biomarker interpretation and clinical utility, although future studies are needed to confirm our findings.

## Supplementary Material

Supplemental data

Supplemental data

Supplemental data

Supplemental data
