# Supplementary material for: Influence of Blood–Brain Barrier Integrity on Brain Protein Biomarker Clearance in Severe Traumatic Brain Injury: A Longitudinal Prospective Study
Source: J Neurotrauma. 2020 May 27;37(12):1381–91. doi: 10.1089/neu.2019.6741 (PMC7249468; doi:10.1089/neu.2019.6741)
Supplement: Supplemental data [file Supp_Fig1.pdf]

Supplementary Data

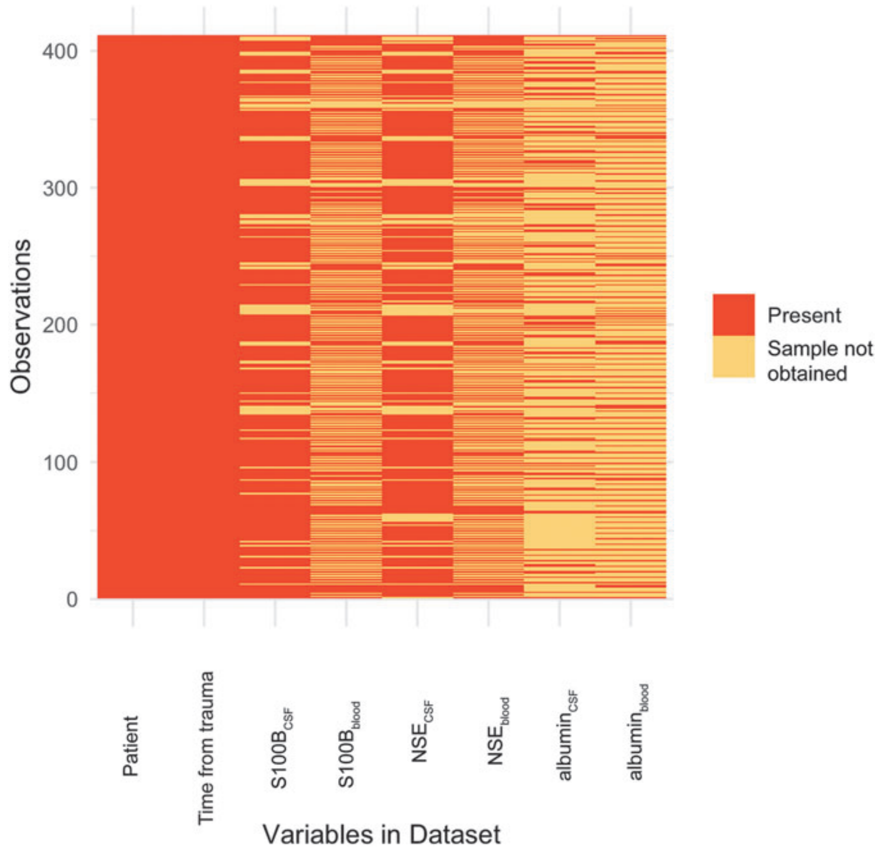

SUPPLEMENTARY FIG. S1. Sample overlap in the data. Depiction of all samples with and without sample overlap across key variables in the data set. Each row/observation in the data set represents one time point. CSF was sampled more frequently than blood, and both biomarkers were sampled more frequently than albumin. CSF, cerebrospinal fluid; NSE, neuron-specific enolase.
