# Supplementary material for: Influence of Blood–Brain Barrier Integrity on Brain Protein Biomarker Clearance in Severe Traumatic Brain Injury: A Longitudinal Prospective Study
Source: J Neurotrauma. 2020 May 27;37(12):1381–91. doi: 10.1089/neu.2019.6741 (PMC7249468; doi:10.1089/neu.2019.6741)
Supplement: Supplemental data [file Supp_Fig2.pdf]

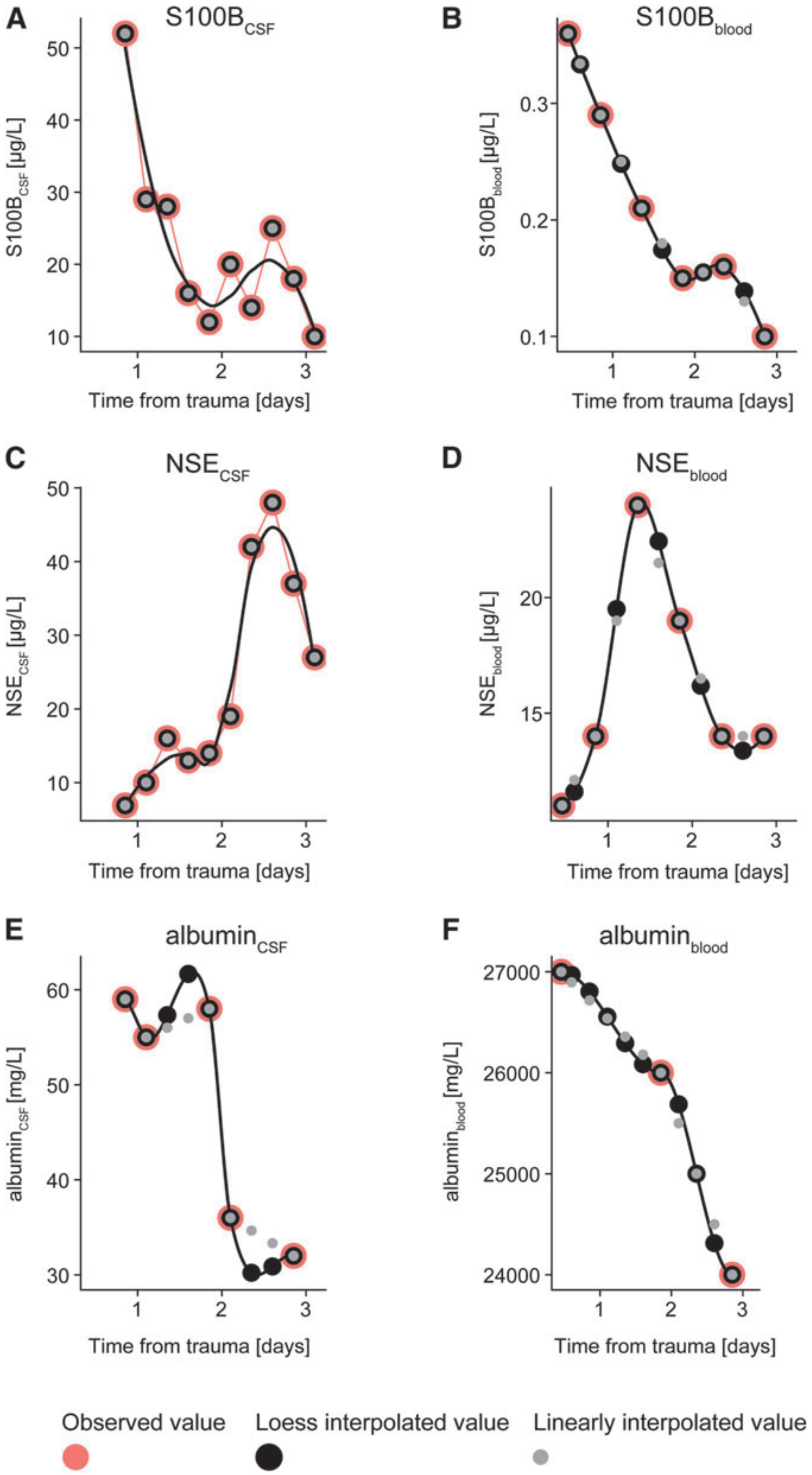

SUPPLEMENTARY FIG. S2. Data interpolation. Comparison of linear and loess interpolation overlayed together with the actually observed values (depicted in red) for one patient. The same patient is depicted across all panels. CSF, cerebrospinal fluid; Loess, locally weighted scatterplott smoother; NSE, neuron-specific enolase.
