# Supplementary material for: Influence of Blood–Brain Barrier Integrity on Brain Protein Biomarker Clearance in Severe Traumatic Brain Injury: A Longitudinal Prospective Study
Source: J Neurotrauma. 2020 May 27;37(12):1381–91. doi: 10.1089/neu.2019.6741 (PMC7249468; doi:10.1089/neu.2019.6741)
Supplement: Supplemental data [file Supp_Table1.pdf]

Supplementary Table S1. Cohort Demographics

Variable	*Type (unit)*	*Value,* n=*16*
Age	Years	45 (25-54)
Male gender	Count (%)	13 (81)
Glasgow Coma Scale		7 (4-7)
Pupil responsiveness	Count (%)	Bilateral responsive: 8 (50)<br>Unilateral unresponsive: 4 (25)<br>Bilateral unresponsive: 4 (25)
Multi-trauma	Count (%)	6 (37.5)
Head AIS	Score (1-6)	1 (minor): 0 (0)<br>2 (moderate): 0 (0)<br>3 (serious): 1 (6.25)<br>4 (severe): 5 (31.3)<br>5 (critical): 10 (62.5)<br>6 (maximum): 0 (0)
Marshall CT Classification	Classes I-VI	I (no visible pathology): 0 (0)<br>II (diffuse injury): 5 (31.3)<br>III (''swelling''): 3 (18.8)<br>IV (''shift''): 1 (6.25)<br>V-VI (''mass lesion''): 7 (43.8)
Brain injury progression	Count (%)	5 (31.3)
Glasgow Outcome Scale (GOS)	Count (%)	GOS1/Dead: 2 (12.5)<br>GOS2/Persistent vegetative state: 0 (0)<br>GOS3/Severe disability: 6 (37.5)<br>GOS4/Moderate disability: 4 (25)<br>GOS5/Good recovery: 4 (25)

Patient demographics, described as summarized data for the whole cohort. Data are depicted as mean ± standard deviation (SD), or median (interquartile range [IQR]), if continuous. Categorical data are depicted as count (%).AIS, Abbreviated Injury Scale; CT, computerized tomography.
